# Local Style Preservation in Improved GAN-Driven Synthetic Image Generation for Endoscopic Tool Segmentation

**DOI:** 10.3390/s21155163

**Published:** 2021-07-30

**Authors:** Yun-Hsuan Su, Wenfan Jiang, Digesh Chitrakar, Kevin Huang, Haonan Peng, Blake Hannaford

**Affiliations:** 1 Department of Computer Science, Mount Holyoke College, 50 College Street, South Hadley, MA 01075, USA; jiang24w@mtholyoke.edu; 2 Department of Engineering, Trinity College, 300 Summit St., Hartford, CT 06106, USA; digesh.chitrakar@trincoll.edu (D.C.); kevin.huang@trincoll.edu (K.H.); 3 Department of Electrical and Computer Engineering, University of Washington, 185 Stevens Way, Paul Allen Center, Seattle, WA 98105, USA; penghn@uw.edu (H.P.); blake@uw.edu (B.H.)

**Keywords:** robot-assisted minimally invasive surgery, surgical tool segmentation, generative adversarial networks, UNet, medical imaging

## Abstract

Accurate semantic image segmentation from medical imaging can enable intelligent vision-based assistance in robot-assisted minimally invasive surgery. The human body and surgical procedures are highly dynamic. While machine-vision presents a promising approach, sufficiently large training image sets for robust performance are either costly or unavailable. This work examines three novel generative adversarial network (GAN) methods of providing usable synthetic tool images using only surgical background images and a few real tool images. The best of these three novel approaches generates realistic tool textures while preserving local background content by incorporating both a style preservation and a content loss component into the proposed multi-level loss function. The approach is quantitatively evaluated, and results suggest that the synthetically generated training tool images enhance UNet tool segmentation performance. More specifically, with a random set of 100 cadaver and live endoscopic images from the University of Washington Sinus Dataset, the UNet trained with synthetically generated images using the presented method resulted in 35.7% and 30.6% improvement over using purely real images in mean Dice coefficient and Intersection over Union scores, respectively. This study is promising towards the use of more widely available and routine screening endoscopy to preoperatively generate synthetic training tool images for intraoperative UNet tool segmentation.

## 1. Introduction

Computer vision and machine learning have experienced rapid development and growth in the last decade. While applications in the medical imaging field are growing, challenges still exist. This manuscript focuses on the use of the UNet, the most widely adopted image segmentation tool for medical imaging. In the context of robot-assisted minimally invasive procedures, accurate surgical tool segmentation is a key component of numerous computer-assisted interventions [1], and may enable robust reconstruction [2] potentially from multiple simultaneous viewpoints [3,4]. Due to the challenges of dynamic deformation, specular reflections, and partial blurriness [5], accurate tool segmentation often requires large surgical image data sets to achieve desirable segmentation performance through data-driven approaches. Such data sets are difficult to acquire due to lack of expert annotation, under-representation of rare conditions, and poor standardization. Furthermore, large surgical image data sets are expensive and oftentimes impractical to acquire from clinical robot-assisted minimally invasive surgeries [6]. Concerns include potential interruption of operation workflow as well as sterilization and data privacy concerns.

### 1.1. UNet in Medical Image Segmentation

The primary purpose of medical image segmentation tasks is to separate different objects or anatomical structures of interest from the rest of the image. These structures often need to be isolated for proper diagnosis of conditions [7] or to remove occluding elements. The use of image segmentation tools, and UNet in particular, is most prominent in the medical imaging field for cardiovascular and brain systems. These anatomical structures are oftentimes imaged using 3D imaging methods, such as with computed tomography (CT) and magnetic resonance imaging (MRI), and thus the UNet has been adapted to a variety of medical imaging modalities.

Brain tumor imaging is most prominently achieved with MRI, and identifying the boundaries of cancerous and healthy tissue is necessary for proper resection of the diseased tissue. Several implementations of UNet have been developed and implemented to successfully segment brain tumor structures in MRI [8,9,10,11]. Similarly, UNet has been used in neural MRI to identify and segment brain lesions [12,13] and to analyze brain development [14,15]. Three-dimensional imaging of the cardiovascular system exhibits a broader range of imaging modalities, including CT and MRI. Lung and pulmonary structures were segmented using the UNet on CT scans [9,16,17,18] and cardiovascular structures with MRI [19,20,21,22]. Three-dimensional UNet segmentation has also been used for segmentation of liver tumors in CT scans [23,24] and MRI [25], prostate and breast cancer in MRI [26,27], multi-organ segmentation from CT [28,29,30], and osteosarcoma [31] and vertebrae [32] from CT. The application of UNet for 2D medical image segmentation covers a range of tasks, including skin lesion segmentation [33], segmentation in microscopy [16,34,35,36], and retinal imaging [37,38,39,40] to name a few. Endoscopic imaging is the modality of interest for 2D image segmentation, with several uses of UNet for these types of images [41,42].

### 1.2. Endoscopic Surgical Tool Segmentation

In robot-assisted minimally invasive surgery, the endoscope is typically the primary imaging modality, and in itself provides a restricted field of view [43]. Understanding the surgical tool location within the endoscope frame and with respect to the surgical anatomy can be of vital importance to provide intelligent, computer-aided assistance or intervention. Tool proximity and localization with respect to anatomy can, for example, inform operation procedure or even be used to isolate imaging of the anatomy for image registration and reconstruction. Several auxiliary sensing methods can be used for tool pose estimation, including robot kinematic information [44] and electromagnetic tracking [45]. However, endoscopic and image-based methods coincide with the operator’s frame of reference and do not require augmentations to the surgical tools or instruments. With sophisticated developments in deep learning, the use of machine vision is an attractive avenue for tool tracking and segmentation. However, the lack of sufficient and established endoscopic image data and standard evaluation or ranking are challenges. Within the available datasets, imaging may be sourced from simulation environments [46], a phantom [47], ex vivo [48,49], or in vivo [50]. Furthermore, the image resolutions, surgical operation type, task conditions (e.g., lighting, smoke, occlusions, and blur), image set size, and tool labeling (e.g., tool tip or bounding box) vary between datasets. Several natural image features can be used for tool detection and segmentation. Image color can provide a natural feature for discrimination [51,52,53], and the image gradient, textures and shapes can also be used [54,55,56]. Early approaches used these features to assist in tool detection and segmentation using support vector machines [54,57] and decision forests [50]. More recently, neural network and UNet-based methods have emerged as promising directions [58,59,60,61,62,63].

#### 1.2.1. Surgical Image Augmentation

Lacking sufficient numbers of real data has been addressed in conventional vision applications with various synthetic image generation approaches [64,65,66]. Unfortunately, most use simple morphological augmentations [67] unsuitable for surgical images, which are rich with the complex and diverse features of real human tissue [68]. The problem remains an open challenge, and several approaches exist in the literature. Surgical simulators, such as the 3D-slicer [69], the RobotiX mentor [70], the dV-Trainer [71], and the AMBF [72], enable users to readily capture large quantities of synthetic training images. However, because these images depict a purely artificial scene, they often lack the visual artifacts and imperfections required to train strong tool segmentation models [73,74]. It is possible to convert the working domain to a synthetic one by training the segmentation network on a large set of synthetic images and real test images converted to synthetic through pre-processing domain transfer [75]. The results are robust, however, the real-to-synthetic domain transfer loses textual cues and details. A similar concept was a adopted with a more readily available target image domain-cadaver images [76]. Labeling was expedited for the cadaver endoscopic imaging by using robot kinematic information [6,58,77,78]. Although both image domains contain some realistic visual details and texture, cadaver data acquisition is expensive.

#### 1.2.2. Rendering via Adversarial Learning

Generative adversarial networks (GANs) [79] have gained traction in the medical imaging field for data generation without explicit need for probability density functions or labeled samples [80]. Compared with traditional training image augmentation methods like scaling, rotation, flipping, and elastic deformation [81], GAN-driven approaches afford the capability to enforce domain specific conditions on the generated images to abide by the surgical workflow, appearance of a particular pathology, or various imaging protocols [82,83]. Conditional GAN-based medical image synthesis research has been explored in numerous medical imaging domains such as CT [84], MRI [85,86,87], Ultrasound [88,89], X-rays [90,91], and retinal fundus imaging [92]. This is useful to synthesize images in uncommon conditions, such as lung nodules along the lung border [93]. However, little work has been done in the endoscopic imaging modality, and even less for surgical tool segmentation. In [63], an image-to-image (I2I) model and robot simulator transferred the realistic style of ex vivo and in vivo RMIS images onto simulated tool images. However, since surgical tool pixels were processed independently of the background, visual effects from reflection, motion blur and tool-tissue interactions were not well modeled. The absence of realistic visual artifacts in the generated images can be addressed by collecting a large number of cadaver images in a similar mock sinus endoscopic surgery setup and conducting cadaver-to-real cross domain image synthesis. This ensures that similar visual effects exist in both the source (cadaver) and target (real) domain [76]. Although realistic images are generated, a generic solution for synthetic endoscopic image generation with pure real dataset remains an open challenge. In another approach, the image synthesis step was bypassed and surgical tool segmentation was implemented directly using GAN-based domain adaptation [94]. The surgical image was the real image domain and the segmented mask was the target domain.

### 1.3. Contributions

This work investigated three novel GAN-driven surgical image augmentation approaches. The best method utilizes the proposed loss function that incorporates both local neural style transfer [95] and a modified CycleGAN [96]-like structure with custom component-level losses. The method was evaluated on a classic tool segmentation model, UNet, with varying levels of synthetic training data composition. To the best of the authors’ knowledge, this work is the first to provide simultaneously:

1. A GAN-driven synthetic surgical endoscopic image generation framework without requiring cross-domain sample images;
2. The development of a custom multi-level loss function that:
  - On the component level, adopts realistic tool textural style, minimizes background content changes and preserves synthetic tool shape;
  - On the image level, incorporates visual artifacts to mimic realistic tool-tissue interaction regions;
3. A systematic guide to evaluate generated synthetic images and identify the ideal composition of real and synthetic training images;
4. Open access of all source code [97,98].

## 2. Methods

Three modified CycleGAN approaches for generating synthetic tool images for endoscopic image segmentation were investigated. All three strategies are improvements upon the baseline tool augmentation method described in Section 2.1. The set of baseline synthetic images S is generated by overlaying real endoscopic surgical backgrounds, the collection of which is denoted $R_{B}$, with a randomly placed artificial surgical tool. The task then is to enhance the baseline artificial tool pixels with realistic appearance. The first strategy performs transfer from the domain of baseline synthetic images S to the domain of real surgical images R through CycleGAN (note: R does not refer to the set of real numbers in this context). The second strategy executes partial GAN application on only the baseline surgical tool pixels. Finally, the third approach utilizes a modified CycleGAN loss design that balances (a) partial style preservation of the background and (b) realistic generation of the tool (visual artifacts and texture) and (c) tool tissue border smoothness of the generated synthetic image.

### 2.1. Baseline Synthetic Image Generation, S

A baseline synthetic image $s_{i}\in S\subset S$ is constructed using two main steps on a preselected real endoscopic background image in $R_{B}$. These steps are

1. Surgical tool augmentation;
2. Circle border pre-processing.

#### 2.1.1. Surgical Tool Augmentation

**Figure 1:**
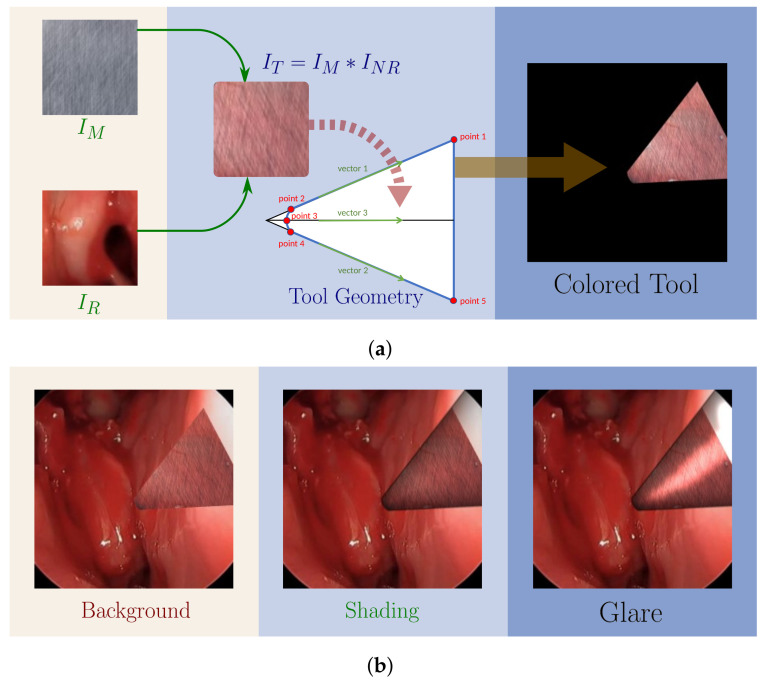
The baseline tool image generation procedure (**a**) tool color (*I_M_* ∗ *I_NR_*) and tool geometry fused to create colored tool patch. (**b**) Post-processing steps by overlaying tool patch on background, shading, and glare modifications.

As depicted in Figure 1a, the synthetic tool shape is defined by 5 key geometric points, which are mostly connected by straight lines. Exceptions exist between key points 2, 3 and 4, which are connected via a 2nd order polynomial. The tools are randomly scaled, shifted and rotated before being placed on each background image. The tool colors $I_{T}$ were rendered from the fusion of a metallic texture image $I_{M}$ and a normalized reflection background image $I_{NR}$.

(1) $$\begin{array}{ccc} I_{T} & = & I_{M}*I_{NR}\\ I_{NR} & = & \alpha \;\frac{I_{R}}{255}+\left(1-\alpha \right) \end{array}$$

where ∗ indicates elemental-wise multiplication, α is an empirically chosen reflection coefficient, and $I_{R}$ represents the reflection background image. A larger α can result in a stronger reflection effect. Once the color is rendered, the tool is overlaid onto the surgical backgrounds in $R_{B}$ with added modifications such as glare and shading to enhance realism as shown in Figure 1b.

#### 2.1.2. Circular Border Pre-Processing

The endoscopic background images in $R_{B}$ used to generate S images are selected from the University of Washington Sinus dataset [49]. Since endoscopes exhibit a circular field of view, each original rectangular background image contains endoscopic information with a circular border contained within the entire image. Between varying endoscopes, the circular borders are inconsistent in size and sometimes off-center. Without first isolating only relevant image sections, unwanted overfit of the tool location based on circle location may occur. To that end, proceeding the surgical tool augmentation step, only the largest square within the circular image is stored as a synthetic baseline image in S.

### 2.2. GAN-Driven Augmentations

The baseline synthetic tool images as depicted in Figure 1 lack sufficient realism. The automated glare and shade additions are incapable of deceiving the human eye. The information used from the surgical background is applied in an inflexible and non-adaptive manner. To improve synthetic tool realism, three modified implementations of the CycleGAN network were developed, which execute transfer between the domain of real surgical images R and the domain of synthetic baseline tool images S.

#### 2.2.1. Naive Global GAN Application (***Strategy I***)

The CycleGAN network consists of four networks: two generators and two discriminators. Given synthetic images {s∈S⊂S} and real surgical scenes {r∈R⊂R} with training domains S and R, the generators G:R→S and F:S→R seek a bijective mapping. Furthermore, they ideally are inverses. The discriminators $D_{S}$ and $D_{R}$ serve the role of two inspectors, where $D_{S}$ and $D_{R}$ evaluate the likelihood that an image belongs to S and R, respectively.

The four networks are trained sequentially with pairs of images $\left(s_{i},r_{i}\right)$, where $s_{i}\in S$ and $r_{i}\in R$. During training, four loss functions are used for optimization, one for each network. First define the following expressions

(2) $$\begin{array}{ccc} f_{\mathrm{cyc}}\left(I_{x},I_{y}\right) & = & \frac{\sum |p_{xi}-p_{yi}|}{N_{x}} \end{array}$$

(3) $$\begin{array}{ccc} f_{\mathrm{gen}}\left(x\right) & = & {\left(x-1\right)}^{2} \end{array}$$

(4) $$\begin{array}{ccc} f_{\mathrm{dis}}\left(x,y\right) & = & {\left(x-1\right)}^{2}+\frac{y^{2}}{2} \end{array}$$

where $I_{x},I_{y}$ are images, $N_{x}$ is the number of pixels in $I_{x}$, $p_{xi}$ and $p_{yi}$ are the *i*th pixels in $I_{x},I_{y}$, respectively, and x,y are real numbers. For each training image pair $\left(s_{i},r_{i}\right)$, loss functions for each network are computed. First define

(5) $$L_{\mathrm{cyc}}=\lambda_{1}f_{\mathrm{cyc}}\left(r_{i},\left(F\circ G\right)\left(r_{i}\right)\right)+\lambda_{2}f_{\mathrm{cyc}}\left(s_{i},\left(G\circ F\right)\left(s_{i}\right)\right)$$

where ∘ is the composition operator, $\lambda_{1},\lambda_{2}$ are heuristically tuned weights. Then the four loss functions for each network are computed as

(6) $$\begin{array}{ccc} L_{G} & = & L_{\mathrm{cyc}}+f_{\mathrm{gen}}\left(\left(D_{s}\circ G\right)\left(r_{i}\right)\right) \end{array}$$

(7) $$\begin{array}{ccc} L_{F} & = & L_{\mathrm{cyc}}+f_{\mathrm{gen}}\left(\left(D_{r}\circ F\right)\left(s_{i}\right)\right) \end{array}$$

(8) $$\begin{array}{ccc} L_{D_{s}} & = & f_{\mathrm{dis}}\left(D_{s}\left(s_{i}\right),\left(D_{s}\circ G\right)\left(r_{i}\right)\right) \end{array}$$

(9) $$\begin{array}{ccc} L_{D_{r}} & = & f_{\mathrm{dis}}\left(D_{r}\left(r_{i}\right),\left(D_{r}\circ F\right)\left(s_{i}\right)\right) \end{array}$$

where $L_{G},L_{F}$ are cycle consistency losses associated with G,F, respectively, and $L_{D_{s}},L_{D_{r}}$ are adversarial losses for $D_{s},D_{r}$, respectively. Adversarial losses characterize the deviation between the distribution of the generated data and that of the original data. On the other hand, the cycle consistency loss ensures that the network has the flexibility to map a set of data to all possible permutations in the target domain [96].

**Figure 2:**
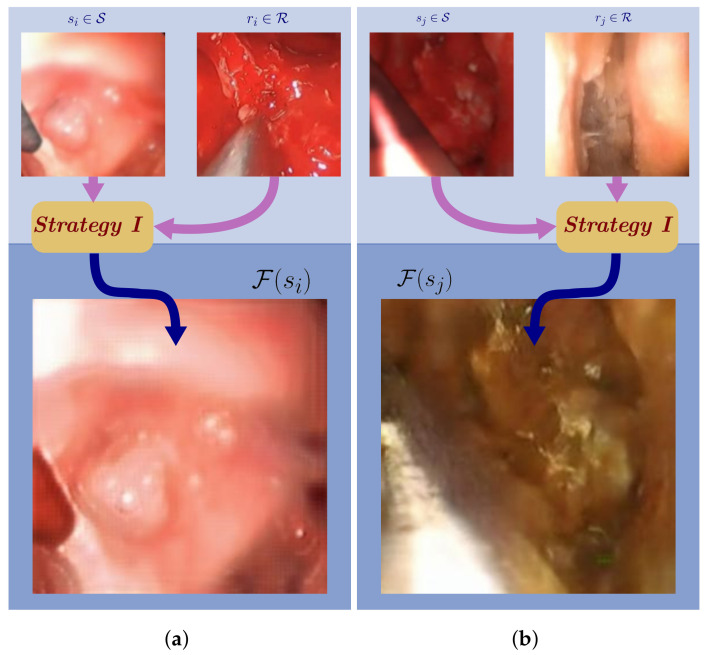
CycleGAN generated synthetic images from ***Strategy I***: (**a**) The generated tool image adopts texture from the background; (**b**) the background adopts texture from the tool.

When training the CycleGAN network, images from each domain were taken in pairs. Figure 2 depicts two example input image pairs ($s_{i}\in S\subset S$, $r_{i}\in R\subset R$), and the resultant synthetic image generated by $F(s_{i})$ after the model is fully trained. Because the approach lacks semantic knowledge of the image, tool pixels and background pixel attributes were often interchanged. In Figure 2a, the final synthetic tool image tool pixels in $F(s_{i})$ inherit tissue color tones, and in Figure 2b the final synthetic tool image background pixels adopt tool color tones. This strategy’s drawbacks made it unacceptable.

#### 2.2.2. Tool Localized GAN (***Strategy II***)

To address the mismatching of color compositions for different semantic elements within the images, the CycleGAN approach was modified to first execute only on isolated synthetic and real tool pixels. For each $s_{i}\in S$, there is an associated binary tool mask ${}_{s}m_{i}$. The isolated tool image from $s_{i}\in S$, call it $s_{t_{i}}$, is then calculated as

(10) $$\begin{array}{c} s_{t_{i}}=s_{i}*{}_{s}m_{i} \end{array}$$

where ∗ denotes pixel-wise multiplication. Isolated tool image for $r_{i}\in R$ are similarly defined as

(11) $$\begin{array}{c} r_{t_{i}}=r_{i}*{}_{r}m_{i}\; \end{array}$$

Let $S_{t}=\left\{s_{t_{i}}|s_{i}\in S\right\}$ and $R_{t}=\left\{r_{t_{i}}|r_{i}\in R\right\}$. The CycleGAN algorithm as described in the previous subsection was then implemented again on the entire images with separately enhanced tool pixels overlaid on background images from $R_{B}$.

**Figure 3:**
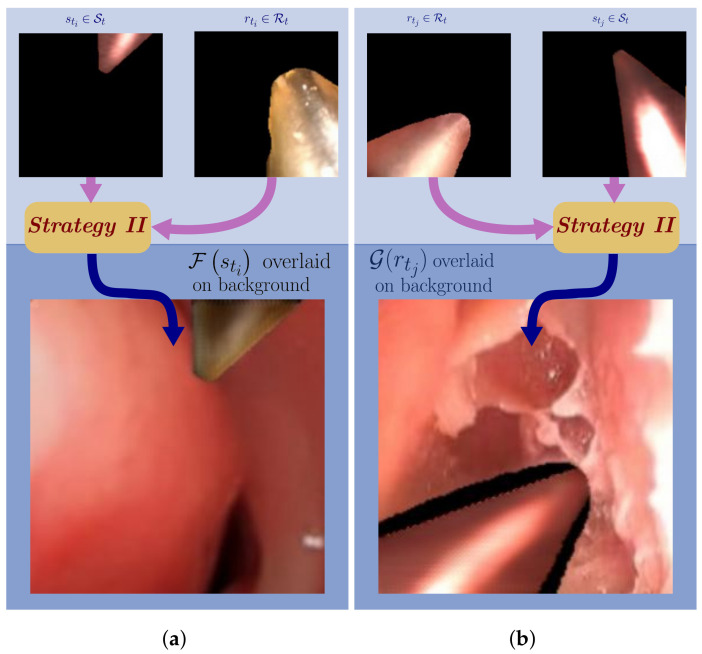
CycleGAN generated synthetic images from ***Strategy II***: (**a**) The tool image was generated ignorant of the background; (**b**) the tool image borders are not preserved.

As depicted in Figure 3, the separately enhanced tool pixels are non-ideal. In Figure 3a, lack of context from the background during isolated tool image training result in tool pixels that do not reflect the surgical scene colors. Furthermore, since morphology of synthetic and real tools vary, when isolated with a black background tool borders are not well incorporated. This is observed in Figure 3b. Because of these faults, this strategy was also deemed unacceptable.

#### 2.2.3. GAN with Partial Style Preservation (***Strategy III***)

The results of the previous two methods demonstrated an instability in the texture, color or shape of the artificial surgical tool. To address these issues, methods for partial style preservation of tool pixels and content preservation of tissue pixels were incorporated while continuing to train the entire image through CycleGAN. This approach aims to minimize textural disparity between generated and real tool pixels while preserving background surgical scene content, processing each of the two semantic portions separately. Style differences between the generated and real tool pixels are minimized within each activation layer.

For content preservation, first define

(12) $$f_{\mathrm{con}}\left(I_{x},I_{y},I_{z}\right)=\frac{\sum \left(1-p_{zi}\right)*\left|p_{xi}-p_{yi}\right|}{N_{x}}$$

where $I_{x},I_{y}$ are images and $I_{z}$ is a binary labeled mask for $I_{x}$, $p_{xi},p_{yi},p_{zi}$ are the *i*th pixels in $I_{x},I_{y},I_{z}$, and ∗ denotes element-wise multiplication.

Then for each of the two generators a content loss is assigned as

(13) $$\begin{array}{ccc} L_{G_{\mathrm{con}}} & = & f_{\mathrm{con}}\left(r_{i},G\left(r_{i}\right),{}_{r}m_{i}\right) \end{array}$$

(14) $$\begin{array}{ccc} L_{F_{\mathrm{con}}} & = & f_{\mathrm{con}}\left(s_{i},F\left(s_{i}\right),{}_{s}m_{i}\right) \end{array}$$

To formulate style preservation, let

(15) $$\begin{array}{ccc} f_{\mathrm{vgg}}\left(I_{x},I_{y}\right) & = & G\left(V\left(I_{x}\right)*V\left(I_{y}\right)\right) \end{array}$$

(16) $$\begin{array}{ccc} f_{\mathrm{sty}}{(}_{x}G,{}_{y}G) & = & \sum_{l<=L}\frac{\omega_{l}{(}_{x}G_{l}-{}_{y}G_{l})*{(}_{x}G_{l}-{}_{y}G_{l})}{4N_{xGl}^{2}} \end{array}$$

where ∗ again indicates element-wise multiplication, $x_{G},y_{G}$ denote the Gramian of images $I_{x},I_{y}$, respectively, *l* iterates through layers, $N_{xGl}$ is the number of elements in xGl, *L* is the number of style layers, the weighting factor for each layer $\omega_{l}=\frac{1}{\left|L\right|}$ in this work, and *V* returns pretrained VGG19 neural network per-layer output.

Recall that CycleGAN is trained sequentially through pairs of images, $\left(s_{i},r_{i}\right)$. Each pair is associated with a pair of binary tool masks ${(}_{s}m_{i},{}_{r}m_{i})$. Then, for each of the two generators, a style loss is assigned as

(17) $$\begin{array}{ccc} L_{G_{\mathrm{sty}}} & = & f_{\mathrm{sty}}\left(f_{\mathrm{vgg}}\left(G\left(r_{i}\right),{}_{r}m_{i}\right),f_{\mathrm{vgg}}\left(s_{i},{}_{s}m_{i}\right)\right) \end{array}$$

(18) $$\begin{array}{ccc} L_{F_{\mathrm{sty}}} & = & f_{\mathrm{sty}}\left(f_{\mathrm{vgg}}\left(F\left(s_{i}\right),{}_{s}m_{i}\right),f_{\mathrm{vgg}}\left(r_{i},{}_{r}m_{i}\right)\right) \end{array}$$

With these parameters defined, the cycle consistency loss functions for training generators G:R→S and F:S→R are modified by augmenting the original expressions in (6) and (7) to style preserving cycle consistency loss functions

(19) $$\begin{array}{ccc} L_{G_{3}} & = & L_{G}+L_{G_{\mathrm{sty}}}+L_{G_{\mathrm{con}}} \end{array}$$

(20) $$\begin{array}{ccc} L_{F_{3}} & = & L_{F}+L_{F_{\mathrm{sty}}}+L_{F_{\mathrm{con}}} \end{array}$$

The style representation of an image is described as the correlation between various filter responses as determined by the image Gramian.

**Figure 4:**
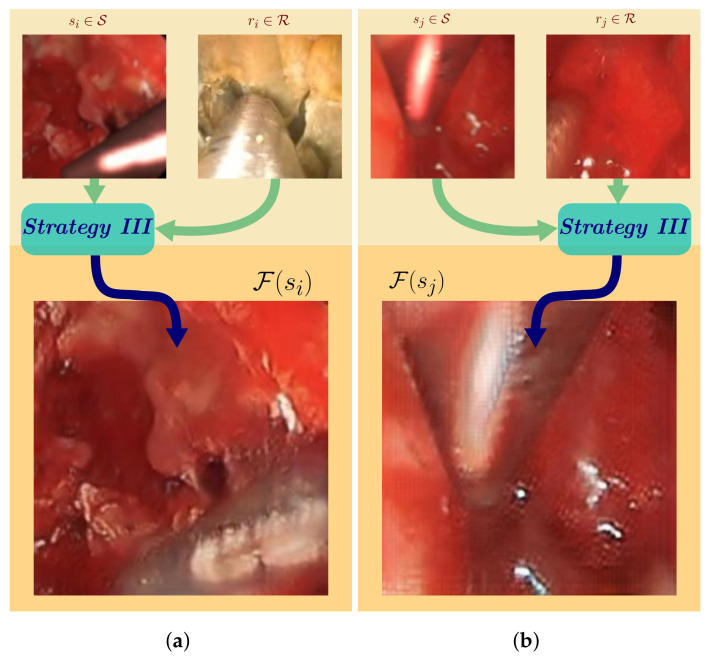
CycleGAN generated synthetic images from ***Strategy III***: (**a**) Tool image style is adopted from the real image, background content is preserved resulting in realistic synthetic image; (**b**) tool border is retained even if *s_j_* and *r_j_* tool shapes vary.

**Figure 5:**
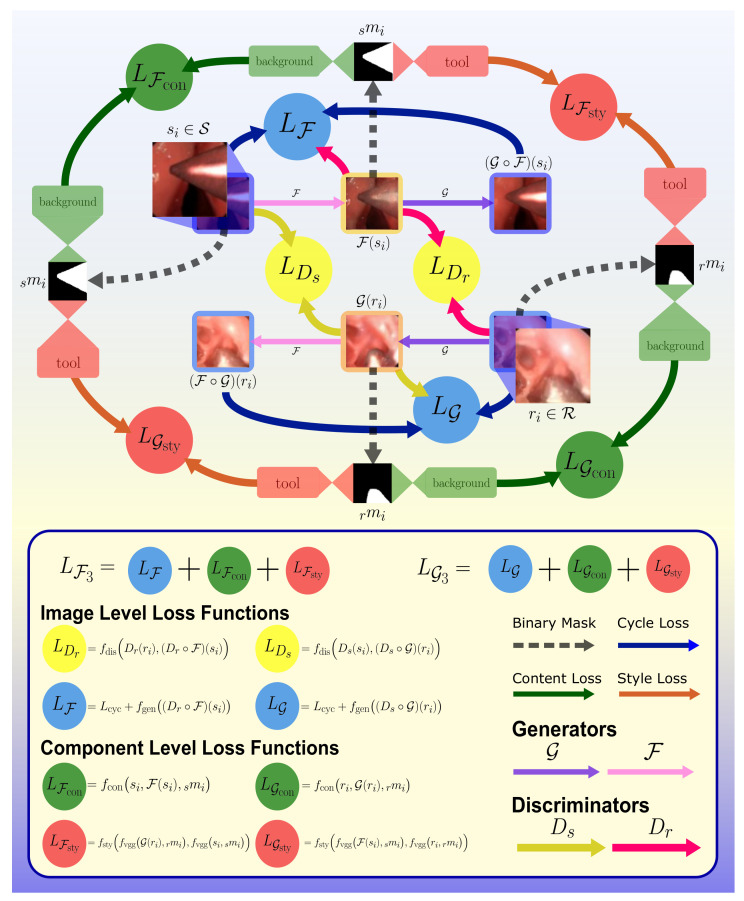
Flowchart diagram depicting ***Strategy III*** loss structure. $L_{D_{r}},L_{D_{s}},L_{F_{3}}$, and $L_{G_{3}}$ represent the loss functions used for $D_{r},D_{s},F$, and G, respectively.

***Strategy III*** is depicted in Figure 4, the structure of which is illustrated in Figure 5. The modified CycleGAN model contains four loss functions: the image level (1) cycle-consistency loss $L_{\mathrm{cyc}}$ and (2) adversarial loss $L_{D_{s}},L_{D_{r}}$ preserve the semantic meaning of the whole image; the component-level (3) style loss of tool $L_{G_{\mathrm{sty}}},L_{F_{\mathrm{sty}}}$ and (4) content loss of tissue $L_{G_{\mathrm{con}}},L_{F_{\mathrm{con}}}$ trace back in the hidden layer activations to perform deep restricted style transfer locally in the surgical tool region of the images while ensuring minimal modifications to the background. The separation of foreground and background were provided in the data set as prior knowledge.

### 2.3. Synthetic Image Verification: Tool Segmentation

Only images generated using ***Strategy III*** were evaluated quantitatively—the two other GAN-driven strategies were investigated but eliminated as viable methods via preliminary tests. To evaluate the utility of the synthetically generated endoscopic images as augmented training data, various combinations of real and synthetic images were used to train a U-Net, a classic surgical tool segmentation network [67]. The network was then tested on real images. These training and testing procedures were designed to answer the following two questions:

**(i)** Does incorporating synthetic images with real training images improve segmentation performance?
**(ii)** Can a large synthetic training set alone be used to train a successful segmentation network to segment test images from R?

Table 1 shows the training set composition for the nine UNet experiments conducted. In each experiment, the UNet was trained on a different proportion of real images from R and GAN-generated synthetic surgical images. The resultant networks were comparatively evaluated on a fixed separate set of real surgical images.

## 3. Experiments

### 3.1. Raw and Baseline Data

The image data used for this study were drawn from the publicly available, de-identified University of Washington Sinus Cadaver/Live Dataset [49,99]. This data set contains a total of 4345 cadaver and 4658 live sinus endoscopic images, denoted R. Each endoscopic image in R is accompanied by a manually labeled annotation, i.e., a pixel-wise labeled mask of the surgical tool.

Section 2.1 describes the baseline synthetic image generation process combining synthetic tool and background images. For this process, a total of 354 images containing only surgical environment pixels and no surgical tools pixels were selected as background baselines. Within this baseline set of images, six categories were defined based on color composition. In total, 30,000 baseline synthetic images (i.e., synthetic tool combined with background image) were generated with uniform color theme distribution, and are denoted as S.

### 3.2. System Workflow

The baseline synthetic images in S, as described in Section 2.1, exhibit realistic surgical tool pixel placement in the spatial and morphological sense. Tool pixel colorization for baseline images in S is uninformed of the background. To better mimic realistic endoscopic data, tool pixels must be modified to reflect the surgical environment as depicted by the surrounding background. This research presents a reliable method for enhancing synthetic baseline images in S into realistic ones with life-like tool pixel colorization.

In general, the approach is GAN-driven with domain transfer between R and S, and several modifications of the CycleGAN code base [96] are experimentally evaluated. Empirically, ***Strategy III*** described in Section 2.2 was found to return the best synthetic image enhancement results. Thus, the following experiments were conducted to quantify the utility of GAN-generated synthetic images using ***Strategy III***. Note that since the CycleGAN approach is bidirectional, a byproduct set of images denoted $G_{S}$ are generated as well, as depicted in Figure 5.

**Figure 6:**
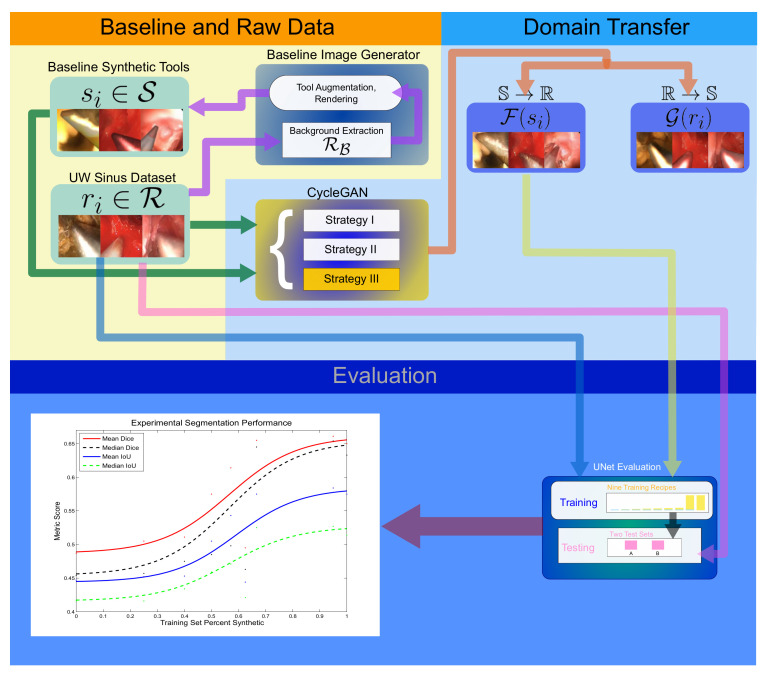
The GAN-driven synthetic image generation framework for endoscopic surgical tool segmentation.

The GAN-driven synthetic image generation procedure ideally extends the scope of otherwise limited endoscopic surgical image data sets with additional training data. If the data are realistic enough, training using the synthetically generated images should result in improved segmentation of real test images. The goal of the experimental tasks was to analytically verify that incorporating the synthetic images into training does indeed increase segmentation performance during testing. This experimental system workflow of this study can be found in Figure 6.

### 3.3. Implementation Details

#### 3.3.1. CycleGAN Image Generation

To train the GAN-driven synthetic image generation model using strategies described in Section 2.2, a total of 5965 training images from each of the two domains, real images R and baseline synthetic S, were randomly selected. Note that training images from domain R were chosen from a random mix of cadaver and live endoscopic images. Meanwhile, the hyperparameters including adaptive base learning rate of 0.0002, max step of 200, pool size of 200, and λ of 10 were heuristically tuned for best performance. The GAN models were then tested on 88 images per domain.

#### 3.3.2. UNet Image Evaluation

The nine experiments as described in Table 1 were evaluated on the same UNet structure. Specifically, the hyperparameters were set to 50 epochs, 100 steps per epoch, 2 batches, Adam’s optimizer learning rate of 0.0001 for optimal results and the binary crossentropy loss was set as the cost function.

The 300 real training images selected from R were consistent across Exp. 1 through 8. Two test sets were set aside to evaluate the UNet performances using the designated training set mixtures. In particular:

- ***Test Set A*** contains 100 random mix of cadaver and live endoscopic images in R;
- ***Test Set B*** contains 100 selected real image frames from R that neighbor the 300 real training images in the University of Washington Sinus Dataset video sequence.

## 4. Results

Two widely accepted and commonly used image segmentation metrics were employed to evaluate performance of the UNet tool segmentation and thus usability of the proposed GAN-driven synthetic image generation framework in surgical tool segmentation tasks:

1. Dice coefficient [100];
2. Intersection over Union (IoU) score [101].

**Figure 7:**
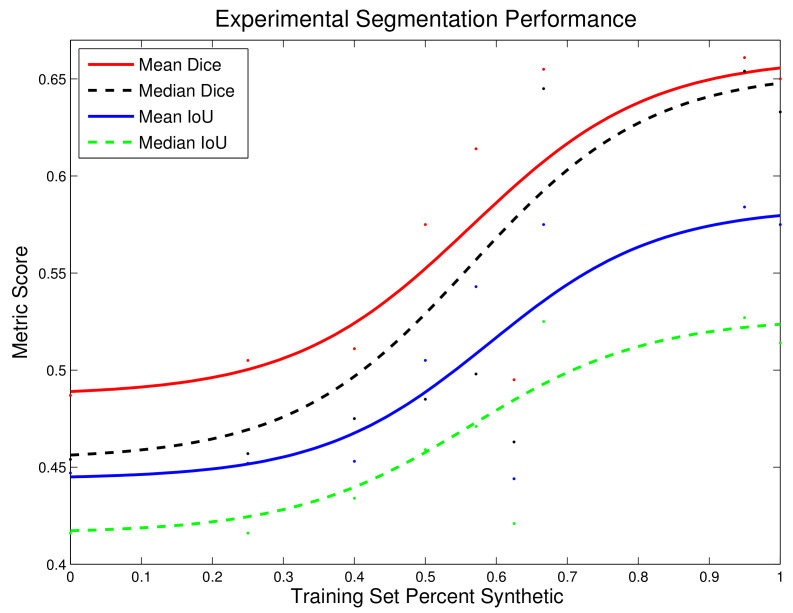
Average Dice and IoU scores for tests on ***Test Set A*** as a greater percentage of the training set is composed of synthetically generated endoscopic images using the presented method. Traces are logistic regression fits to the data.

The average Dice and IoU scores segmenting the test set with varying training set synthetic composition is shown in Figure 7.

**Figure 8:**
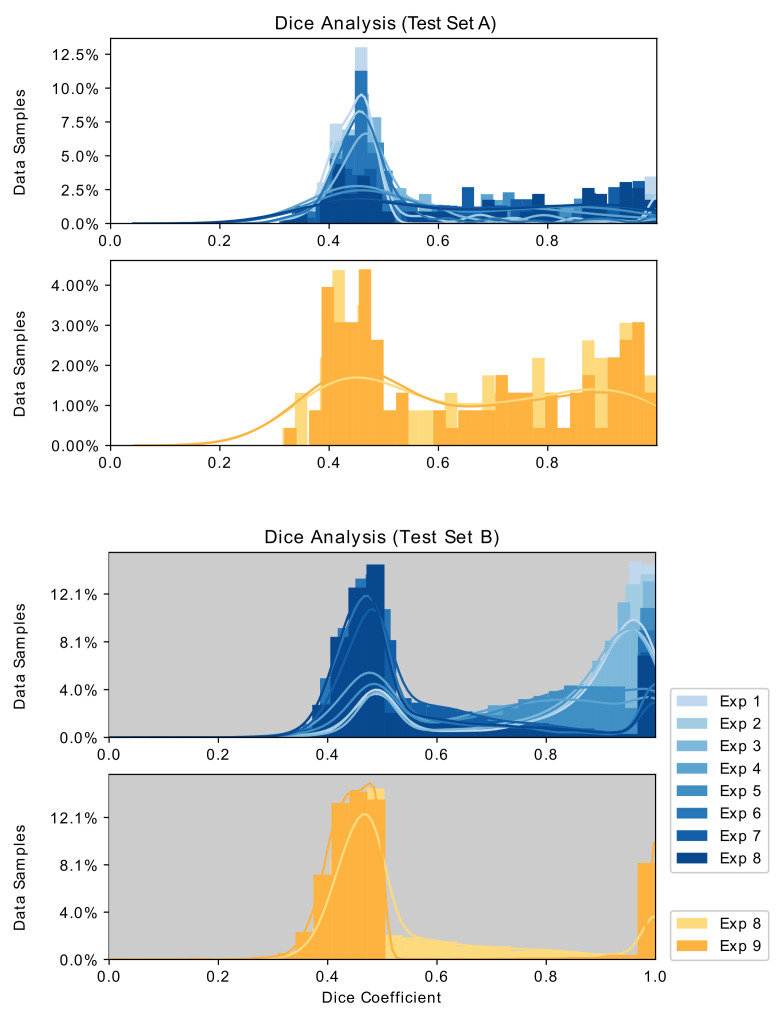
Histograms and distribution curves of the Dice coefficients of Exp. 1–8 and Exp. 8,9 evaluated on ***Test Set A*** and ***Test Set B***.

**Figure 9:**
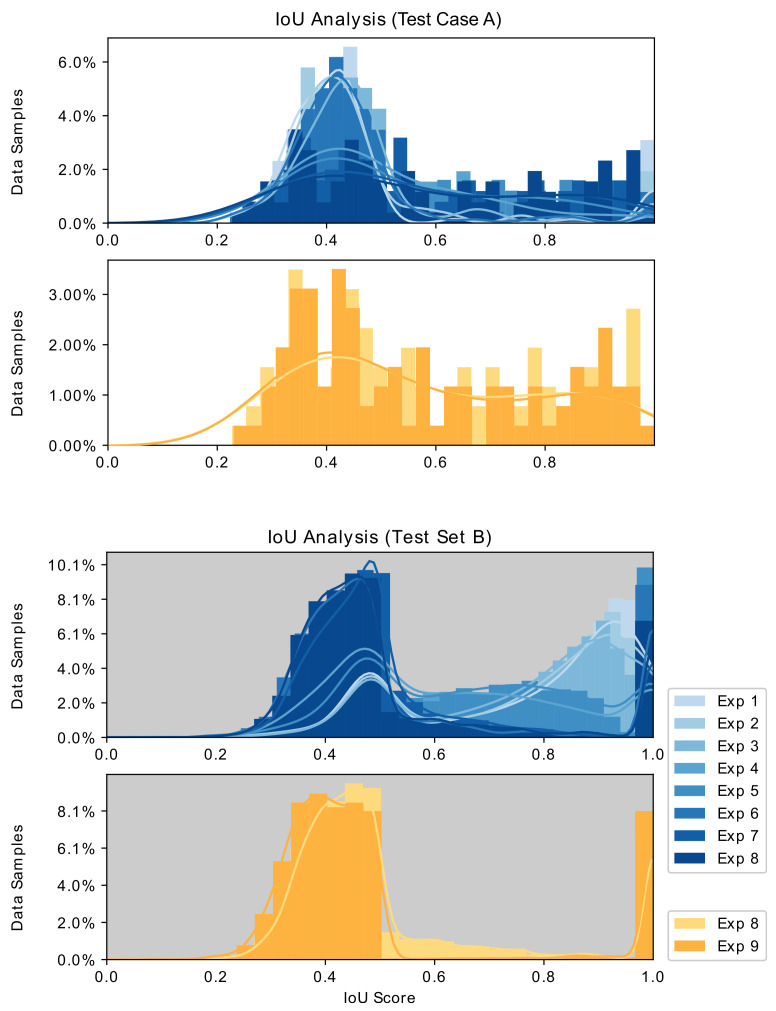
Histograms and distribution curves of the IoU scores of Exp. 1–8 and Exp. 8,9 evaluated on ***Test Set A*** and ***Test Set B***.

**Figure 10:**
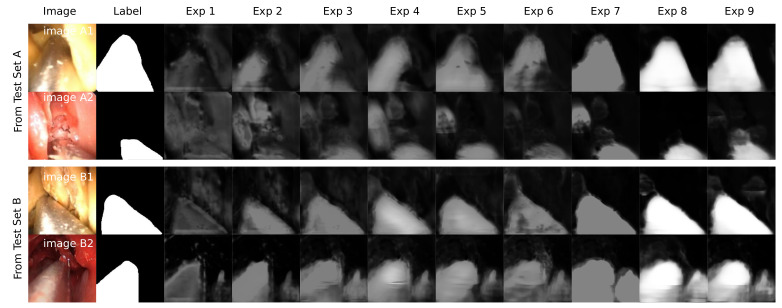
Sample predictions from ***Test Set A*** and ***Test Set B*** in Exp. 1–9. The prediction confidence is greater in later experiments. Images A1 and A2 show poor results in early experiments. Image B2 demonstrates a phantom 2nd tool falsely detected from Exp. 7 onward.

Figure 8 and Figure 9 depict the evaluation histograms for Dice Coefficient and for IoU, while Figure 10 shows sample predictions across Exp. 1–9. The mean and median scores are summarized in Table 2.

These results indicate that the composition in Exp. 1 resulted in the least training loss and the best performance on ***Test Set B***, while Exp. 8 achieved the best scores on ***Test Set A***.

## 5. Discussion

The experimental results summarized in Table 2, Figure 8 and Figure 10 lead to several observations about the training image compositions and segmentation performances on the two test sets. These are described below.

### 5.1. Dilution of Real Training Images

***Test Set A*** is an unbiased analysis of the UNet model segmentation performance with random endoscopic data samples, while ***Test Set B*** provides an indication of model performances for segmenting images similar to the real training images. From Exp. 1 through Exp. 9, the training set was augmented with increasing proportion of randomly selected synthetic images. As such, greater variance is introduced into the training set, and hence the trained model is more generalizable. The segmentation performance enhances with increased synthetic data augmentation using ***Test Set A***. On the other hand, the 300 real training image samples are increasingly diluted within the training set with increasing Exp. number. Thus, the trained models perform progressively worse on test images similar to the original real training images, i.e., ***Test Set B***.

### 5.2. Overfitting with Small Training Set

In Table 2, overfitting is observed in the first few experiments, as indicated by lower training loss, and good segmentation performance on ***Test Set B*** but poor segmentation with ***Test Set A***. The overfitting is also observed in the blue histograms in Figure 8, as darker histograms perform better with ***Test Set A*** but worse with ***Test Set B***. This overfitting issue unfortunately is a common problem in data-driven semantic medical image segmentation tasks when the training size is too small.

Exp. 1–8 also indicate several performance trends with increased synthetic composition of training data:

- From Exp. 5 onward, a consistent negative correlation between training loss and ***Test Set A*** scores is observed;
- From Exp. 6 onward, training loss is monotonically decreasing, ***Test Set A*** metrics monotonically increasing;
- From Exp. 7 onward, ***Test Set A*** performance surpassed that of ***Test Set B***.

These observations indicate that, in this experiment using 300 real training images, the addition of 400 (Exp. 5) or more synthetic images to the training set effectively neutralizes detrimental variance introduced by the GAN-driven synthetic images. Furthermore, the addition of 500 (Exp. 6) or more synthetic images steadily enhances general endoscopic tool segmentation performance. Meanwhile, interpreting ***Test Set B*** scores as an indicator of the training performance, Exp. 7 marks the point when the validation surpasses training performance, and therefore when overfitting is resolved.

### 5.3. Training with Synthetic Images

In Exp. 1–8, the training set contained a fixed number of real images from R, with increasing addition of synthetic images. The results show with increased proportion of synthetic training data, an overall enhancement of segmentation performance is observed when testing with arbitrarily selected real images, i.e., ***Test Set A***. Furthermore, with a purely synthetic training set, results are promising in Exp. 9 with performance of up to 0.650 Dice Coefficient, which is comparable to that of Exp. 8, the best performing training composition for ***Test Set A***.

### 5.4. Purely Synthetic Training

The results from this work indicate that GAN-driven synthetic images provided enhanced surgical tool segmentation performance. However, the presence of real images in Exp. 8 resulted in superior performance to models trained with purely synthetic data, Exp. 9. This was true for both ***Test Set A*** and ***Test Set B*** measured performance, as observed in the gold histograms in Figure 8. This suggests that a large quantity of purely synthetic images generated per ***Strategy III*** does not completely replace the value of even a small number of real training images.

### 5.5. Implications

To evaluate the practical feasibility of enhancing tool segmentation performance in robot-assisted surgical procedures using partial-synthetic training sets, nine UNet models were trained with different proportions of the synthetic and real images designed to address two queries of interest as described in Section 2.3.

Based on the numerical results in Section 4 and the aforementioned analysis in Section 5, insight is gained with regard to answering these two questions:

**(i)** The addition of the generated synthetic images to a small set of real images can indeed enhance segmentation performance. To maximize this improvement, two conditions should be followed:
  (a) The test images share a broader variance than the set of available real training images;
  (b) The number of synthetic images is sufficiently large.
**(ii)** A large set of purely synthetic training images as generated in this work does not eliminate the benefit of real surgical training images. With large purely synthetic training set, performance is satisfactory.

## 6. Conclusions

In summary, this research showcased a promising GAN-driven approach for generating reliable synthetic training data for surgical tool segmentation. As depicted in Table 1, Exp. 1–8 were designed to train the UNet segmentation network with the same, high-cost real endoscopic images and with varying number of low-cost synthetic endoscopic images generated via the proposed method. Exp. 9 used a training set with purely synthetic images. As shown in Table 2, the best mean Dice and IoU scores for random test images, i.e., ***Test Set A***, were achieved with a training set consisting of 95% synthetic images. Figure 7 shows that addition of the generated synthetic data tends to increase performance. To summarize, for ***Test Set A*** mean Dice and IoU scores of:

- 0.487 and 0.447 for 0% synthetic training set;
- 0.661 and 0.584 for 95% synthetic training set

were observed, respectively. This corresponds to a 35.7% and 30.6% increase. The results are promising and suggest that the proposed method can enhance segmentation results with limited availability of real training data. The availability of even a small amount of real training data is still beneficial.

In this exploratory, baseline experiment, the addition of synthetic training images generated by the novel framework were evaluated by incorporating increasing proportion of synthetic data to the training set, which originated from a publicly available set of endoscopic surgical images. A widely used and accepted network, the UNet, was used identically across experimental conditions to execute the segmentation, and results suggest that the generated synthetic data are indeed useful and benefit tool segmentation performance. Comparable methods in the domain of endoscopic tool segmentation synthetic image generation are not readily evaluated on the same baseline dataset. In other works, methods may have been quantitatively evaluated only on a private dataset, or the source code was not made available for public use to replicate and compare.

The designed experiments do provide a guideline for systematically quantifying the usability and requirements of synthetic training data for other applications. Enhancing surgical tool segmentation can enable broader research efforts in multicamera surgical reconstruction [102,103] within the context of vision-based force estimation [104,105,106] and other robot-assisted medical procedures.

Two possible directions to advance this study are of interest:

(a) Compare the proposed method with other synthetic endoscopic surgical image generation approaches and combinations thereof;
(b) Automate the GAN-driven synthetic image generation process to adaptively optimize the number and distribution of training images based on training and validation sets.

## Figures and Tables

**Table 1:** Experimental training set compositions.

Exp	Real Images	Synthetic Images	Total Training Set
1	**300**	0	300
2	**300**	100	400
3	**300**	200	500
4	**300**	300	600
5	**300**	400	700
6	**300**	500	800
7	**300**	600	900
8	**300**	**5665**	5965
9	0	**5965**	5965

Note: The fixed number of real training images in Exp. 1–8 (**blue**) were designed to aid in addressing research question (i), whereas Exp. 8–9 utilize training sets heavily composed of synthetic data (**gold**) to aid in addressing (ii).

**Table 2:** Experimental segmentation performance results.

Metric	Training Loss	Dice Coeff.			
Exp	Mean	Mean	Median	Mean	Median
1	0.130	0.487	0.454	0.447	0.416
		0.824	0.920	0.776	0.856
2	0.202	0.505	0.457	0.452	0.416
		0.821	0.913	0.772	0.844
3	0.283	0.511	0.475	0.453	0.434
		0.825	0.904	0.771	0.830
4	0.336	0.575	0.485	0.505	0.459
		0.701	0.719	0.637	0.596
5	0.289	0.614	0.498	0.543	0.471
		0.746	0.791	0.681	0.676
6	0.332	0.495	0.463	0.444	0.421
		0.576	0.486	0.525	0.452
7	0.325	0.655	0.645	0.575	0.525
		0.584	0.499	0.524	0.473
8	0.308	0.661	0.654	0.584	0.527
		0.566	0.483	0.513	0.449
9	0.321	0.650	0.633	0.575	0.514
		0.524	0.455	0.487	0.418

The scores displayed with white background denote results from ***Test Set A***, and those with gray tinted background indicate results from ***Test Set B***. The best performances within each test set and metric are specified with blue text.
